# TonEBP suppresses adipogenesis and insulin sensitivity by blocking epigenetic transition of PPARγ2

**DOI:** 10.1038/srep10937

**Published:** 2015-06-04

**Authors:** Jun Ho Lee, Hwan Hee Lee, Byeong Jin Ye, Whaseon Lee-Kwon, Soo Youn Choi, Hyug Moo Kwon

**Affiliations:** 1 School of Life Sciences, Ulsan National Institute of Science and Technology, Ulsan, Republic of Korea

## Abstract

TonEBP is a key transcription factor in cellular adaptation to hypertonic stress, and also in macrophage activation. Since TonEBP is involved in inflammatory diseases such as rheumatoid arthritis and atherosclerosis, we asked whether TonEBP played a role in adipogenesis and insulin resistance. Here we report that TonEBP suppresses adipogenesis and insulin signaling by inhibiting expression of the key transcription factor PPARγ2. TonEBP binds to the PPARγ2 promoter and blocks the epigenetic transition of the locus which is required for the activation of the promoter. When TonEBP expression is reduced, the epigenetic transition and PPARγ2 expression are markedly increased leading to enhanced adipogenesis and insulin response while inflammation is reduced. Thus, TonEBP is an independent determinant of adipose insulin sensitivity and inflammation. TonEBP is an attractive therapeutic target for insulin resistance in lieu of PPARγ agonists.

Tonicity-responsive enhancer binding protein (TonEBP), also known as nuclear factor of activated T cells 5 (NFAT5), belongs to the Rel family of transcription factors along with nuclear factor κB (NFκB) and NFAT1 to NFAT4^1^,^2^. Members of this family share the Rel-homology domain of ~250 amino acids involved in DNA binding. TonEBP was originally identified as the central regulator of transcriptional response to hypertonic stress^1^,^3^,^4^,^5^. Recent studies have revealed that TonEBP is also involved in the pro-inflammatory activation of macrophages by promoting expression a host of pro-inflammatory genes in response to Toll-like receptor 4 activation^6^, as well as by stimulating the activity of NFκB^7^,^8^. Genetic haploinsufficiency of TonEBP is associated with reduced inflammation leading to nearly complete prevention of rheumatoid arthritis and atherosclerosis in mouse models^9^,^10^. Thus, relatively moderate reduction in TonEBP expression results in surprisingly dramatic suppression of inflammatory diseases making TonEBP an attractive therapeutic target.

Adipose tissues play a central role in energy homeostasis^11^,^12^,^13^. Under conditions of excess caloric intake, expansion of adipose tissue is a healthy way of storing excess lipid because excess lipid deposition in cells other than adipocytes causes lipotoxic side effects^14^,^15^,^16^. Adipose inflammation is involved in various aspects of the adipose tissue expansion. On one hand, adipocyte inflammation is required for adipogenesis and healthy adipose tissue expansion. Adipocyte-specific reduction in inflammation results in reduced adipogenesis and limited adipose tissue expansion in combination with increased ectopic lipid accumulation in response to excess caloric intake^17^. On the other hand, obesity is often associated with higher number of pro-inflammatory macrophages within the adipose tissue leading to chronic adipose inflammation and insulin resistance^18^,^19^,^20^,^21^,^22^,^23^.

Given the role of TonEBP in various inflammatory diseases, our long-term goal is to determine the function of TonEBP in adipose tissue expansion and insulin resistance. We began the quest by examining the role of TonEBP in adipogenesis and adipocyte inflammation. Here we report that TonEBP suppresses the epigenomic transition of the peroxisome proliferator-activated receptor gamma 2(PPARγ2) promoter and adipogenesis while promoting adipocyte inflammation.

## Results

### TonEBP suppresses adipocyte differentiation in association with PPARγ downregulation

**Figure 1:**
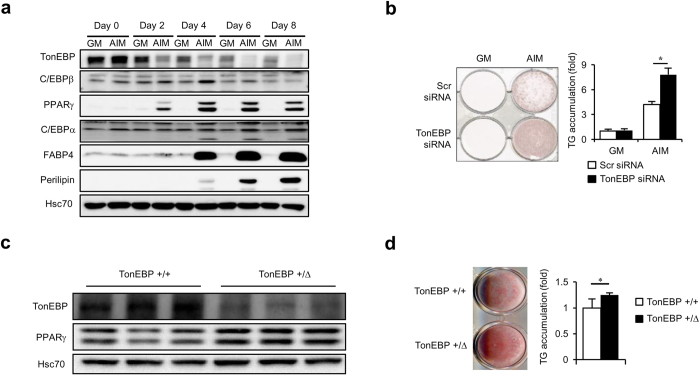
TonEBP expression decreases during adipogenesis, and knockdown or genetic deficiency of TonEBP expression enhances adipocyte differentiation (**a**) 3T3-L1 cells were cultured in growth medium (GM) or adipogenesis inducing medium (AIM) for up to 8 days as indicated and immunoblotted for various proteins named on the left. (**b**) Cells were transfected with TonEBP-targeted siRNA or scrambled (Scr) siRNA followed by culture in GM or AIM for 6 days, and stained with Oil-Red-O and the stain was extracted and measured to assess intracellular TG. Mean + SD, n=4. (**c** and **d**) Stromal vascular fractions were isolated from subcutaneous fat pads of *TonEBP*^*+/Δ*^ mice and their *TonEBP*^*+/+*^ littermates, and cultured for 5 days in modified AIM. Immunblots (**c**) and TG contents (**d**) are shown. Mean + SD, n = 3. *p < 0.05.

To understand the function of TonEBP in adipocyte differentiation, we first examined TonEBP expression at several time points during adipocyte differentiation. 3T3-L1 cells were switched to adipogenesis inducing medium (AIM) for up to 8 days. Adipogensis was evident as indicated by pronounced expression of peroxisome proliferator-activated receptor γ (PPARγ - both PPARγ1 and PPARγ2 are shown as double bands), fatty acid binding protein 4 (FABP4), and perilipin (Fig. 1a), and accumulation of triglycerides (Fig. 1b). During this period, TonEBP levels decreased markedly compared to non-differentiating cells, i.e., those cells kept in growth medium (GM) (Fig. 1a). The decrease was evident on day 2 and continued up to day 8.

We investigated how adipocyte differentiation was affected after manipulating TonEBP expression. First, we examined the effects of TonEBP over-expression using adenovirus in 3T3-L1 cells. Over-expression of TonEBP followed by switch to AIM resulted in a decreased accumulation of triglycerides and reduced expression of PPARγ’s (see Supplementary Fig. S1), indicating that adipocyte differentiation was blunted. Reversely, knock-down of TonEBP using siRNA before switching to AIM resulted in an increased accumulation of triglycerides (Fig. 1b).

We further examined the effect of TonEBP using pre-adipocytes obtained from the stromal vascular fractions of mouse subcutaneous fat fads as described in Methods. We explored the *TonEBP*^*+/Δ*^ mice (heterozygotes) because they display TonEBP haplo-deficiency^5^. As expected, differentiated adipocytes from the *TonEBP*^*+/Δ*^ mice showed reduced TonEBP expression compared to those from *TonEBP*^*+/+*^ littermates (Fig. 1c). The *TonEBP*^*+/Δ*^ cells showed elevated expression of PPARγ’s (Fig. 1c) and higher triglyceride content (Fig. 1d) consistent with the suppressive effects of TonEBP on adipocyte differentiation.

**Figure 2:**
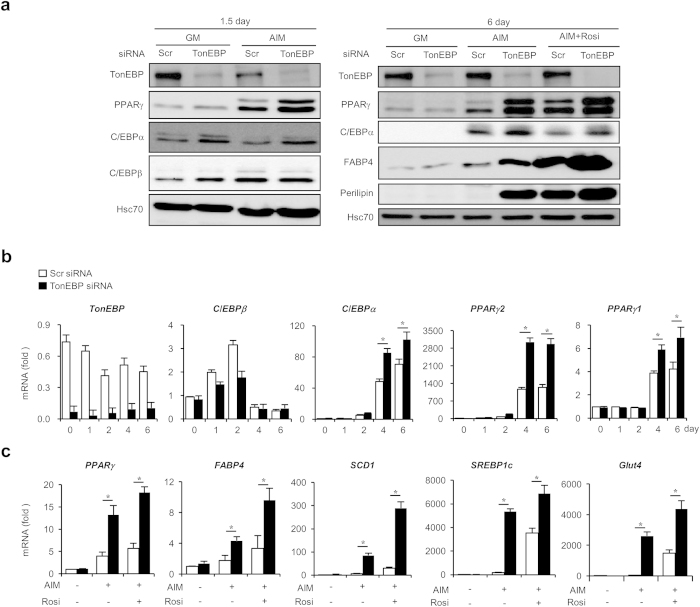
Enhanced expression of adipogenic proteins and genes in response to TonEBP knockdown. (**a**) 3T3-L1 cells were transfected with scrambled or TonEBP-targeted siRNA followed by culture in GM or AIM for 1.5 or 6 days. Where indicated, 1 μM rosiglitazone (Rosi) was added. Immunoblotting was performed as in Fig. 1. (**b**) Cells transfected with scrambled (open bars) and TonEBP-targeted siRNA (filled bars) were cultured in AIM for up to 6 days as indicated. Quantitative PCR was performed for mRNA for TonEBP and adipogenic genes and expressed in fold over 0 day. (**c**) Cells transfected with siRNA were cultured for 6 days in GM or AIM without or with rosiglitazone. Quantitative RT-PCR was performed for adipogenic genes and Glut4. Mean + SD, n = 4. *p < 0.05.

Expression of PPARγ’s increased both on 1.5 and 6 days in AIM after TonEBP knockdown, which responded to rosiglitazone treatment by further enhancing their expression along with adipogenic proteins such as FABP4 and perilipin (Fig. 2a). The increases in protein expression were associated with parallel increase in their mRNA’s as well as mRNA’s for other adipogenic genes - stearoyl-CoA desaturase 1 (SCD1), sterol regulatory element-binding protein 1c (SREBP1c), and glucose transporter 4 (glut4) (Fig. 2b and c) - demonstrating that stimulation of transcription was involved in the process. Since PPARγ is a key transcription factor involved in the upregulation of adipogenic genes^24^ and expression of CCAAT-enhancer-binding protein β (C/EBPβ) was not affected in response to TonEBP knockdown (Fig. 2a), we examined PPARγ further. Transcriptional activity of PPARγ measured by a PPAR-responsive element reporter was enhanced by TonEBP knockdown as well as by rosiglitazone (see Supplementary Fig. S2). In addition, the PPARγ proteins induced by TonEBP were found mostly in the nucleus (see Supplementary Fig. S2) further demonstrating that they were transcriptionally active. Enhanced expression of PPARγ by TonEBP knockdown and rosiglitazone treatment synergistically stimulated adipogenic and lipogenic expression. These data suggest that TonEBP suppress adipogenesis by way of reducing PPARγ expression.

During adipose tissue expansion caused by calorie overload, adipogenesis is stimulated by hypertrophied adipocytes leading to adipocyte hyperplasia^25^. The bone marrow-derived mesenchymal stem cell can be committed into adipogenic fate and it is the critical step for adipogenesis *in vivo*^25^. We asked whether TonEBP had suppressive effects on adipogenesis in human bone marrow-derived mesenchymal stem cells. Consistent with other data here, TonEBP knockdown promoted the adipogenic commitment of these cells (see Supplementary Fig. S3) further supporting the role of TonEBP in the adipogenesis under physiological conditions.

### TonEBP suppresses insulin signaling in adipocytes

**Figure 3:**
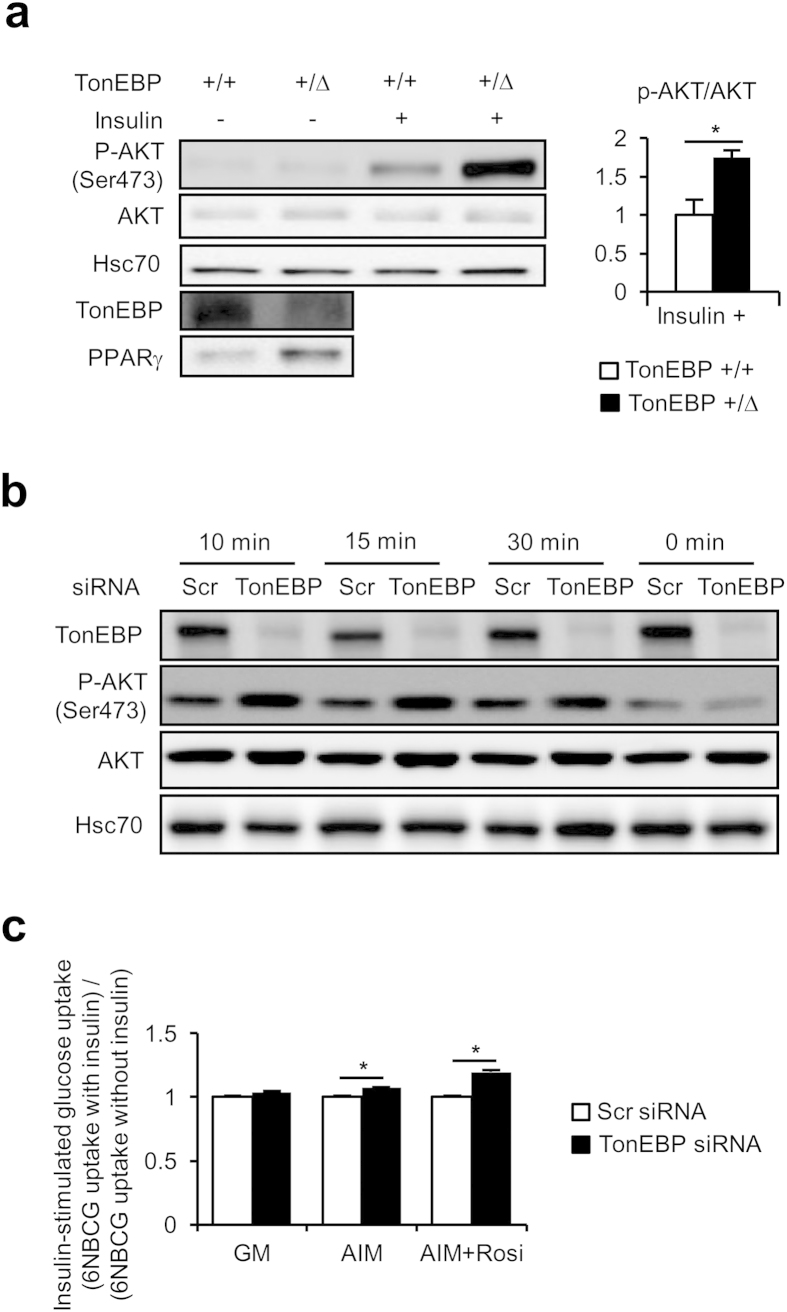
Enhanced insulin response in TonEBP deficiency. (**a**) Primary adipocytes were isolated from epididymal fat pads of *TonEBP*^*+/Δ*^ mice and their *TonEBP*^*+/+*^ littermates. Some of the adipocytes were treated with insulin as indicated before they were immunoblotted for AKT and p-AKT, as well as TonEBP and PPARγ. The ratio of p-AKT/AKT is shown in Mean + SD, n = 3. (**b**) 3T3-L1 transfected with scrambled or TonEBP-targeted siRNA were cultured in AIM. After serum starvation, the cells were treated without (0 min) or with insulin for up to 30 min as indicated, followed by immunoblotting for AKT and p-AKT. (**c**) Cells transfected with siRNA’s were cultured for 5 days in GM or AIM without or with rosiglitazone. The cells were serum-starved before a 30 min uptake of 6-NBDG in the absence or presence 100 nM insulin was measured. The ratio of uptake in the presence of insulin over the absence is shown Mean + SD, n = 3. *p < 0.05.

Adipocyte differentiation is accompanied by enhanced capability to transport glucose and lipid into the cells^13^,^17^,^25^. As adipocytes are central regulator of glucose and lipid homeostasis, their insulin responsiveness is critical^21^,^22^,^23^. We asked whether TonEBP affected insulin signaling in adipocytes. First, we examined insulin-responsive AKT phosphorylation at serine 473. We explored adipocytes obtained from animals from the TonEBP haplo-deficient, *TonEBP*^*+/Δ*^ mice. Again, adipocytes from the heterozygotes displayed reduced TonEBP expression, and enhanced PPARγ2 expression, compared to adipocytes from their *TonEBP*^*+/+*^ littermates (Fig. 3a). More importantly, insulin-stimulated AKT phosphorylation was >70% higher in *TonEBP*^*+/Δ*^ adipocytes compared to *TonEBP*^*+/+*^ adipocytes. Also, in 3T3-L1 cells whose TonEBP had been knocked down before they were induced to differentiate into adipocytes, insulin-stimulated AKT phosphorylation was elevated (Fig. 3b) indicating enhanced insulin signaling. This observation demonstrates that variations in the level of TonEBP expression negatively influence insulin signaling in adipocytes.

We next analyzed insulin-stimulated glucose uptake in differentiated 3T3-L1 cells. Initial rate of glucose uptake was measured using a nonhydrolyzable fluorescent glucose named 6-(*N*-(7-nitrobenz-2-oxa-1,3-diazol-4-yl)amino)-6-deoxyglucose (6-NBDG). As shown in Fig. 3c, insulin-stimulated glucose uptake was significantly elevated in those cells whose TonEBP had been knocked down regardless of the absence or presence of rosiglitazone. The elevated glucose uptake is consistent with increased Glut4 mRNA observed in these cells (Fig. 2c). In sum, TonEBP suppresses insulin signaling in adipocytes in such a way that lower TonEBP expression is associated with elevated glucose uptake and AKT phosphorylation.

### TonEBP suppresses the PPARγ2 promoter by blocking epigenetic activation

The process of adipocyte differentiation involves a complex network of transcription factors leading to expression of the master regulator PPARγ. AIM contains insulin which promotes expression of SREBP1c, 3-isobutyl-1-methylxanthine (IBMX) which activates cAMP-responsive element-binding protein (CREB) *via* elevating cellular cAMP concentrations, and dexamethasone which promotes CCAAT-enhancer-binding protein δ (C/EBPδ) expression *via* activation of glucocorticoid receptor. All of these transcription factors are involved in the transcriptional stimulation of the PPARγ gene.

In order to understand how TonEBP suppressed adipogenesis, we examined the effects of deleting individual AIM components. Deleting insulin, IBMX, or dexamethasone dramatically reduced adipogenesis based on cellular accumulation of triglycerides (see Supplementary Fig. S4) and mRNA levels of PPARγ and FABP4 (see Supplementary Fig. S4). On the other hand, the stimulation by TonEBP knockdown was maintained in every case indicating that TonEBP action was largely independent of these factors. Since PPARγ mRNA was elevated in response to TonEBP knockdown, we asked whether TonEBP directly suppressed the PPARγ promoter.

**Figure 4:**
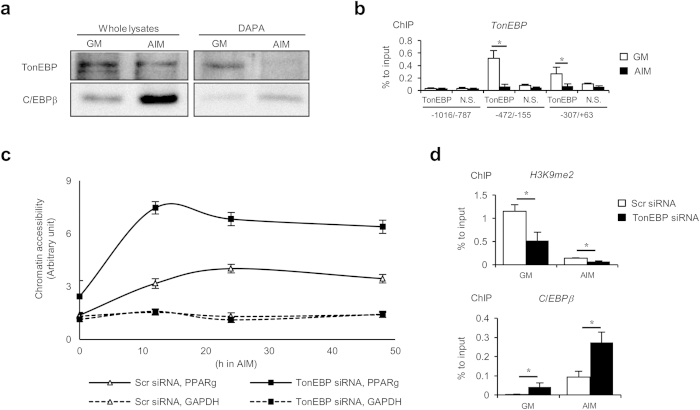
TonEBP binds to the PPARγ2 promoter, and blocks histone methylation and chromatin accessibility. (**a**) 3T3-L1 cells were cultured in GM or AIM for 18 h, and then, DNA affinity precipitation assay (DAPA) was performed with probes containing sequence of 0.5 kb PPARγ2 promoter (see Fig. 6a). The whole lysates and the DAPA products were analyzed with immunoblotting. (**b**) Chromatin immunoprecipitation (ChIP) analysis was performed using cells cultured in GM (open bars) or AIM (filled bars) for 12 h with anti-TonEBP serum (TonEBP) or normal serum (N.S.). Precipitated DNA samples were analyzed by q-PCR with primer pairs for various regions of the PPARγ2 promoter indicated at the bottom. Mean + SD, n = 3. *p < 0.05. (**c**) Cells transfected with scrambled or TonEBP-targeted siRNA in GM were switched to AIM for up to 48 h, as indicated. Nuclei were prepared, and half of them were treated with micrococcal nuclease while the other half was treated without the enzyme. Genomic DNA was isolated and analyzed by q-PCR using primer pairs covering -360/-155 of the PPARγ2 promoter, and -273/-203 of the GAPDH promoter. Chromatin accessibility was calculated from (amount of PCR product in undigested sample) / (amount of PCR produce in digested sample). Error bars representing SD are not shown because they are smaller than the marks representing means (n = 3). (**d**) Cells were transfected with siRNA’s as indicated in GM. The cells were then cultured for 12 h in GM or AIM followed by ChIP analyses using anti-H3K9me2 and anti-C/EBPb antibodies. Precipitated DNA samples were analyzed by q-PCR with primers covering -360/-155 region of the PPARγ2 promoter. Mean + SD, n = 3. *p < 0.05.

PPARγ2, which is the upper band of the doublet shown in Fig. 1a^26^, is the predominant form of PPARγ expressed in adipocytes^26^ as shown in (Fig. 3a). A critical early step in adipogenesis is the activation of the PPARγ2 promoter. This is initiated by epigenetic transition in which de-methylation of Histone H3 allows chromatin opening leading to the initial recruitment of C/EBPb^27^,^28^,^29^. We asked whether TonEBP played a role in the epigenetic transition. We started by examining TonEBP interaction to the promoter region even though we did not find potential TonEBP DNA binding sites. Using DNA affinity precipitation assay (DAPA) using a probe covering the proximal 0.5 kb upstream the PPARγ2 gene, we observed a strong TonEBP binding in undifferentiated cells, i.e., 3T3-L1 cells in GM (Fig. 4a). 18 h after switch to AIM, the binding decreased dramatically coincident with the chromatin remodeling^30^. This dissociation of TonEBP from the promoter took place without significant decrease in TonEBP expression level suggesting that the dissociation was regulated at a post-translational level. The TonEBP binding was confirmed by chromatin immunoprecipitation (ChIP) analyses (Fig. 4b). The ChIP data also revealed that the binding was limited within 360 bp upstream of the gene as little binding was observed upstream, for example 1016 to 787 bp upstream. Next, we examined chromatin opening of the proximal promoter region by analyzing sensitivity to micrococcal nuclease to calculate chromatin accessibility as described in Fig. 4c. Chromatin accessibility increased in 12 h after switch to AIM which was maintained up to 48 h. This “chromatin opening” was associated with reduced di-methylation of lysine 9 in Histone H3 and increased C/EBPb binding (Fig. 4d, open bars). Interestingly, chromatin accessibility was dramatically higher in those cells whose TonEBP had been knocked down in association with further decrease in the di-methylation of Histone H3 and potent increase in the C/EBPb binding. Taken together, the data in Fig. 4 demonstrate that TonEBP binding to the proximal region of the PPARγ2 promoter is associated with Histone H3 di-methylation and prevention of C/EBPb binding. The bound TonEBP dissociates from the promoter at early stages of adipogenesis. Knockdown of TonEBP enhances de-methylation of Histone H3, chromatin accessibility and C/EBPb binding.

**Figure 5:**
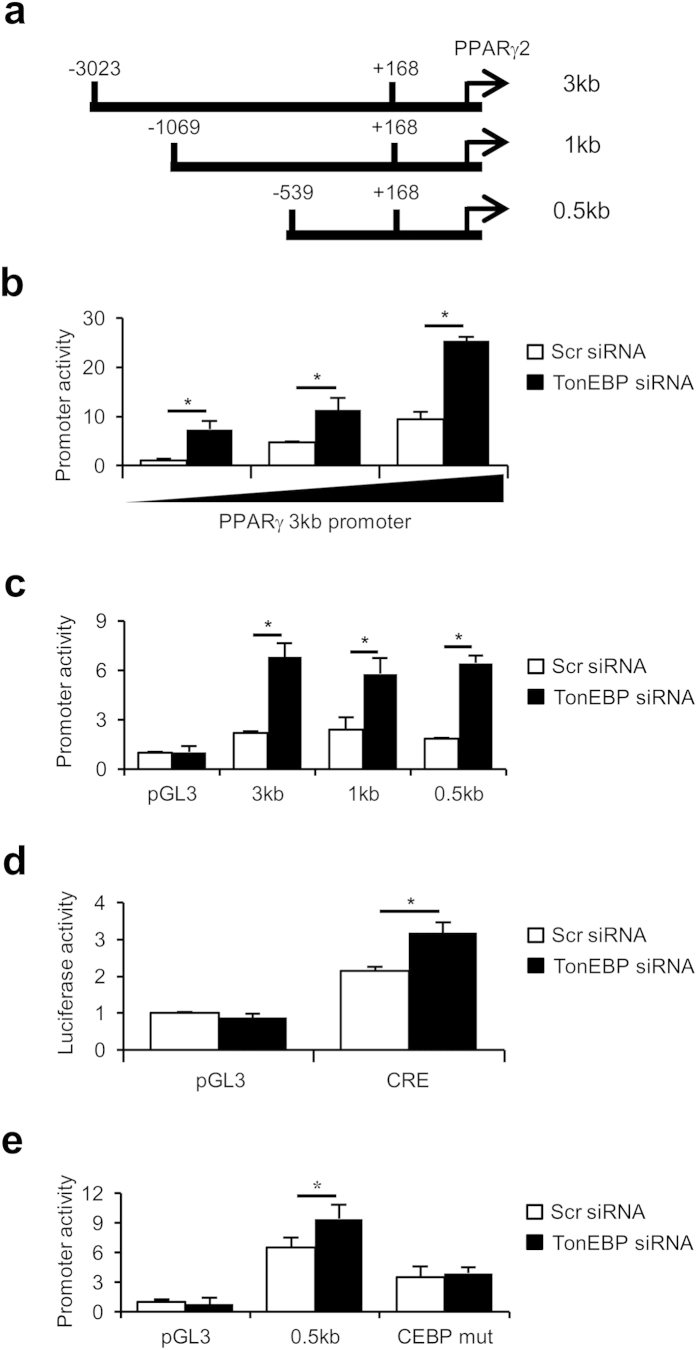
TonEBP deficiency stimulates the PPARγ2 promoter in a C/EBP-dependent manner. (**a**) Schematic representation of the PPARγ2 promoter fragments cloned from mouse genomic DNA. Each of them was cloned in pGL3 to create a luciferase reporter. (b to e) 3T3-L1 cells transfected with scrambled (Scr, open bars) or TonEBP-targeted siRNA (filled bars) were cultured for 2 days in AIM. Then, they were transfected with increasing amounts of the 3 kb promoter reporter (**b**) or promoter-less pGL3 or each of the 3 promoters (**c**). In (**d**), a C/EBP-reporter construct (CRE) or pGL3 was transfected. In (**e** ), the 0.5 kb promoter construct was mutated to remove CEBP binding sites to create ‘CEBP mut,’ as described in Methods. Luciferase activity is shown in Mean + SD, n = 4. *p < 0.05.

We cloned fragments of mouse genomic DNA covering nucleotide position −3023 to +168 relative to the transcription start site of PPARγ2, −1069 to +168, and−530 to +168 as shown in Fig. 5a. These DNA fragments were individually cloned upstream of a promoter-less luciferase reporter (pGL3). Cells transfected with the longest reporter (PPARγ 3 kb promoter) displayed luciferase activity in a dose-dependent manner (Fig. 5b). Interestingly, the promoter activity was stimulated by TonEBP knockdown in all three constructs (Fig. 5c) indicating that TonEBP-responsive transcriptional suppression was localized within 0.5 kb upstream of the transcription start site. C/EBPα is one of the transcription factors recruited to the PPARγ2 promoter subsequent to the initial binding of C/EBPb^26^,^27^. C/EBPα expression was enhanced in response to TonEBP knockdown (Fig. 2) most likely as a result of the increased expression PPARγ which stimulates the C/EBPα promoter^26^,^27^. Since there is a C/EBP binding site in the proximal region of the PPARγ2 promoter, we measured C/EBP activity and found that it was increased in response to TonEBP knockdown (Fig. 5d). As expected, the stimulation of the PPARγ2 promoter construct by TonEBP knockdown was dependent on the C/EBP binding site (Fig. 5e).

The data in Figs. 4 and 5 demonstrate two sites of TonEBP action in the suppression of the PPARγ2 promoter – epigenetic suppression of the locus by direct binding, and indirect inhibition of C/EBPα expression *via* suppression of PPARγ2 expression. The major site is clearly the epigenetic suppression of the promoter locus by way of Histone H3 methylation. When TonEBP expression is lowered, the epigenetic opening of the promoter in response to adipogenic signal is greatly enhanced.

### Reduced pro-inflammatory cytokine expression in response to TonEBP knockdown despite the increased adipogenesis

**Figure 6:**
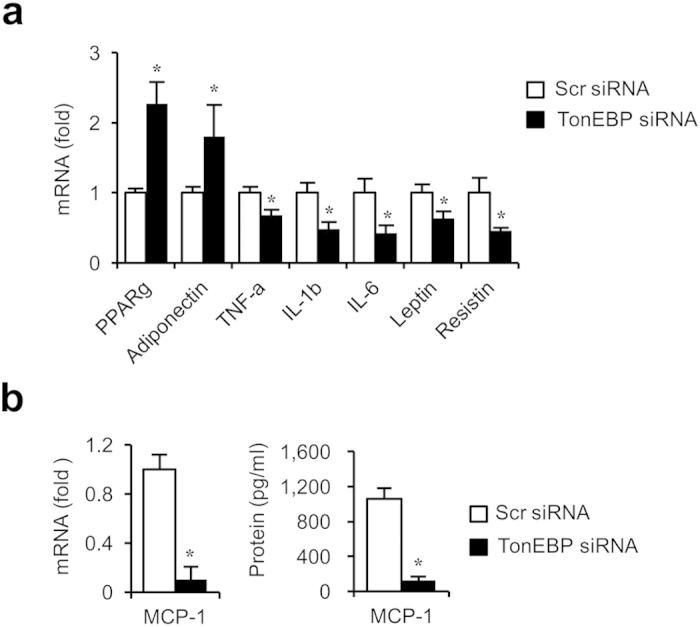
TonEBP stimulates adiponectin expression but suppresses pro-inflammatory cytokines. (**a**) 3T3-L1 transfected with scrambled (open bars) or TonEBP-targeted siRNA (filled bars) were cultured for 6 days in AIM. Quantitative RT-PCR was performed form mRNA’s shown. (**b**) MCP-1 mRNA and MCP-1 in the culture media were measured. Mean + SD, n = 4. *p < 0.05 vs. corresponding scrambled siRNA.

One of the main features of adipogenesis is the upregulation of pro-inflammatory cytokines^31^. In visceral fats, adipocyte inflammation is required for healthy adipose tissue expansion and remodeling during calorie overload^17^. On the other hand, chronic adipose tissue inflammation drives insulin resistance^18^,^19^,^20^,^21^,^22^,^23^. In macrophages, TonEBP promotes expression of pro-inflammatory genes by directly binding to their promoters^6^. We examined expression of inflammation-related genes in adipocytes differentiated from 3T3-L1 cells. As expected from the elevated PPARγ expression, adiponectin mRNA increased in response to TonEBP knockdown (Fig. 6a). On the other hand, mRNA levels of a host of pro-inflammatory genes – TNFα, IL-1b, IL-6, leptin, and resistin – decreased in response to TonEBP knockdown. In addition, expression and secretion of monocyte chemotactic protein-1 (MCP-1), a chemokine for immune cell infiltration to adipose tissues, also reduced dramatically (Fig. 6b). TonEBP deficiency is expected to lessen inflammation in the adipose tissues due to the combined effects of elevated adiponectin and suppressed pro-inflammatory cytokines and chemokines.

## Discussion

Given the pro-inflammatory actions of TonEBP in settings of rheumatoid arthritis^12^ and atherosclerosis^13^, our motivation of this study was to investigate the role of TonEBP in obesity and insulin resistance because inflammation is critically involved^32^,^33^,^34^. As a first step in this direction, we focused on adipocytes and adipogenesis. To our surprise, we find that TonEBP suppresses PPARγ2 expression by binding to the proximal region of the PPARγ2 promoter. This binding is associated with di-methylation of the lysine 9 in Histone H3 and limited chromatin access. In response to adipogenic signals, TonEBP dissociates from the promoter leading to opening of the locus in association with reduced Histone H3 methylation and recruitment of C/EBPb. Reduced TonEBP expression results in a profound enhancement of PPARγ2 expression leading to increased adipogenesis and insulin signaling.

Our data also demonstrate that TonEBP promotes adipose inflammation as indicated by higher adiponectin expression in combination with lower pro-inflammatory genes in response to TonEBP deficiency. Thus, under conditions of TonEBP deficiency adipogenesis is promoted despite reduced inflammation. This may not necessarily contradict the requirement of adipose inflammation for adipogenesis^17^ perhaps because sufficient level of inflammation was maintained in TonEBP deficient conditions: Except for MCP-1, most of pro-inflammatory genes displayed moderate suppression by TonEBP deficiency.

In view of the role of inflammation in adipose insulin resistance^18^,^19^,^20^,^21^,^22^,^23^,^32^,^33^,^34^, the actions of TonEBP seem puzzling: While TonEBP promoted inflammation, it suppressed insulin sensitivity in adipocytes. Disconnect between obesity and insulin resistance has been observed in patients^35^,^36^. For example, about one in four obese individuals are metabolically healthy as judged by insulin resistance and systemic inflammation^37^. Individual variations in the level of TonEBP expression can potentially contribute to such disconnect. One can envision an obese individual with low level of TonEBP expression. This individual might have healthy insulin sensitivity despite the obesity. We reported previously that diabetic nephropathy is associated with ~50% higher TonEBP expression among patients with type 1 diabetes^38^ demonstrating that there are substantial and meaningful individual variations in the level of TonEBP expression. In this regard, we are intrigued by the improved adipocyte insulin sensitivity in animals with TonEBP haploindeficiency. This observation raises the possibility that variations in TonEBP expression level lead to individual variations in adipose tissue insulin sensitivity and may contribute to disconnect between obesity and insulin resistance in certain individuals.

As discussed above, deficiency of TonEBP due to siRNA-mediated knockdown or mutant TonEBP allele elevates PPARγ expression and improves insulin signaling in combination with reduced inflammation. As such, TonEBP deficiency mimics many aspects of synthetic PPARγ activators such as thiazolidinediones^39^,^40^,^41^,^42^,^43^. TonEBP provides an attractive therapeutic target for insulin resistance in lieu of thiazolidinediones.

## Methods

### Cell culture and transfection

3T3-L1 pre-adipocytes cell line from American Type Culture Collection (ATCC) were maintained in Dulbecco’s modified Eagle’s medium (DMEM, Hyclone, Logan, UT, USA) supplemented with 10% Bovine Calf Serum (BCS, Gibco-BRL, Gaithersburg, MD, USA) with penicillin-streptomycin (Hyclone). When the cells grow at the 70% of confluence, cells were transfected with TonEBP siRNA or control scrambled siRNA using lipofectamine RNAiMAX (Invitrogen, Carlsbad, CA, USA) following the manufacturer’s instructions. For induction of adipocyte differentiation, AIM including 1 μM dexamethasone, 0.5 mM isobutylmethylxanthine, and 1 μM insulin (MDI) in DMEM supplemented with 10% Fetal Bovine Serum (FBS, Gibco-BRL) was used as described^44^. Human bone marrow derived mesenchymal stem cell (hBMSC) purchased from Cambrex /Lonza (Walkersville, MD, USA) were maintained in DMEM supplemented with 10% FBS. At the 90% of confluence, cells were transfected with same procedure as 3T3-L1. Adipocyte differentiation was induced by AIM with indomethacin (100 μM) as described^45^. For adenoviral overexpression, 3T3-L1 cells were infected with adenovirus expressing TonEBP or β-galactosidase at the 70% of confluence before switching to AIM.

### Mice

All the methods involving live mice were carried out in accordance with the approved guidelines. All experimental protocols were approved by Institutional Animal Care and Use Committee of the Ulsan National Institute of Science and Technology (UNISTACUC-12-15-A).

### Stromal vascular fraction (SVF) isolation

SVF’s were isolated from 6-week-old male *TonEBP*^*+/Δ*^ mice and their *TonEBP*^*+/+*^ littermates. Subcutaneous fat pads were dissected and minced in Krebs-Ringer buffer supplemented with HEPES (pH7.4), 2% bovine serum albumin, 5 mM D-glucose, 100 mM adenosine, and 2 mg/ml type II collagenase. They were incubated at 37 ˚C with gentle shaking at 100 rpm for 45 min. After centrifugation at 500 g for 10 min, pellets were resuspended and incubated with RBC lysis buffer (ACK lysing buffer, Gibco-BRL) for 10 min. After filtration through a 70 μm filter, the cells were cultured for 2 days in complete DMEM/F12 (Lonza Walkersville, MD). Adipogenesis was induced in a modified AIM containing insulin (10 μg/ml), dexamethasone (1 μM), IBMX (0.5 mM), Indomethacin (125 μM), T3 (1 nM), and rosiglitazone (1 μM) for 2 days, followed by 3 days in DMEM/F12 supplemented with insulin (10 μg/ml) and T3 (1 nM).

### Western blot analysis and nuclear fraction extraction

Cells were washed with PBS and lysed in 10 mM Tris (pH 7.5), 150 mM NaCl, 1 mM EDTA, 1 mM EGTA, 1% Triton X-100, protease inhibitor, 1 mM sodium orthovanadate, and phosphatase inhibitor cocktail for 30 min at 4 ˚C. Isolated cell extracts were separated on SDS-PAGE and transferred to nitrocellulose membrane (Whatman, Clifton, NJ, USA). After blocking nonspecific binding sites with 5% nonfat milk at RT for 1 hr, membranes were incubated with primary antibodies overnight at 4 ˚C. Antibodies to C/EBPα, C/EBPβ, PPARγ, FABP4, Perilipin, p-AKT(Ser473), and AKT were obtained from Cell Signaling Technologies (Berkeley, CA, USA) and antibodies to Hsc70 were obtained from Rockland (Gilbertsville, PA, USA).The immune complexes were detected with horseradish peroxidase-conjugated secondary antibodies. Protein detection was performed by using enhanced chemiluminescence (Pierce, Rockford, IL, USA). Nuclear fraction was extracted using a nuclear extract kit (Pierce) according to the manufacturer’s recommendation.

### Oil-Red-O staining

Differentiated adipocytes are washed by PBS, fixed with 4% paraformaldehyde for 1 hour, washed with PBS, and then stained with 0.6% Oil red O dye in isopropanol and water (6:4) for 1 hour at 4 ˚C followed by washing with PBS. Then, the stained cells were de-stained with isopropanol and optical density was measured by spectrometry at 500 nm wavelength. Optical density of each sample was normalized by that of scrambled siRNA-transfected cell cultured in growth medium.

### qRT-PCR analysis

Total RNA was isolated using Trizol reagent (Invitrogen). Reverse transcription was performed with 4μg of total RNA, and resultant cDNA were subjected to quantitative time PCR for TonEBP, C/EBPβ, C/EBPα , PPARγ1/2, FABP4, SREBP-1c, SCD1, ACC, adiponectin, and pro-inflammatory genes and a loading control, 36B4. Primers used are described in supplementary table S1.

### Isolation of primary adipocytes

Primary adipocytes were isolated from 3-month-old male animals as described^46^. Briefly, epididymal fat pads were minced in Krebs-Ringer buffer (5 ml/g tissue) supplemented with 25 mM HEPES (pH7.4), 2% bovine serum albumin, 5 mM D-glucose, 100 mM adenosine, and 2 mg/ml type II collagenase. They were incubated at 37 ˚C with gentle shaking at 100 rpm for 45 min. After centrifugation at 500 rpm for 30 sec, adipocyte fraction was separated. Adipocytes were incubated with Krebs-Ringer-HEPES buffer supplemented with 50 nM insulin for 10 min with gentle shaking before immunoblot analyses.

### Glucose uptake assay and Insulin signaling

Glucose uptake rate was measured as described^47^. Briefly, cells were serum started for 2 h before they were incubated for 30 min in low glucose DMEM containing 20 μM 6-NBDG without or with 100 nM insulin. The cells were lysed and fluorescence was measured using λ_ex_ = 466 nm and λ_em_ = 540 nm. To assess insulin signaling, cells were starved for 18 h, and then, incubated for 30 min without or with 100 nM insulin. The cells were immunoblotted for AKT and p-AKT, as described above.

### Promoter and Reporter assay

Mouse PPARγ2 promoter fragments (Fig. 5a) were inserted into pGL3 (Promega, Madison, WI, USA). C/EBP binding site in 0.5 kb PPARγ2 promoter construct was mutated using the following primer - AGT(GCA)ATTTTAAAAAAA(GC)AAT (wild type sequence in parenthesis). At the 2 day after differentiation, cells were transfected for 6 hours with PPARγ2-pGL3 and renilla luciferase plasmids. After 24 hours of post-induction using DMEM with insulin (1 μM), luciferase activity was measured. 3x PPAR-luc reporter, PPAR responsive element (PPRE), were from J.B. Kim^48^. At the 4 days after differentiation, cells were transfected with PPRE reporter plasmid and renilla luciferase plasmids. After post-inducing adipogenesis using DMEM with insulin (1 μM), luciferase activity was measured. Three CEBP responsive elements (CRE, underlined below) reporter plasmid was produced by cloning double stranded CRE oligonucleotide (AGATCTGTTGCGCAAGTGGAGGTTGCGCAAGTGGCAGGTTGCGCAAGCTCGAG) into pGL3-basic as described^49^. CRE reporter plasmid was transfected after 2 day of adipogenic induction. After 6 hours, additional adipogenic induction was done by MDI cocktails. 24 hours after additional induction of adipogenesis, luciferase reporter activity was measured.

### DNA affinity precipitation assay (DAPA)

Cells were washed with PBS and lysed in 20 mM Tris, pH 7.5, 150 mM NaCl, 1% Triton X-100, protease inhibitor, 1 mM sodium orthovanadate, and phosphatase inhibitor cocktail for 30 min at 4 °C. 0.5 mg/ml of protein was incubated with 30 nM biotinylated oligonucleotide probe overnight at 4 °C in 7.2 mM Tris [pH7.5], 16 mM HEPES [pH 7.5], 4% glycerol, 166 mM NaCl, 0.4 mM EDTA, 0.8 mM MgCl_2_, and 0.28% Triton X-100. Protein-DNA complexes were isolated using pre-cleared streptavidin-agarose bead and analyzed by western blotting

### Chromatin accessibility assay by qPCR

Chromatin accessibility assay by q-PCR was performed using MNase described as previously with minor modification^50^. Washed cells were lysed in cold NP-40 lysis buffer (10 mM Tris-HCl (pH 7.4), 10 mM NaCl, 3 mM MgCl_2_, 0.5% NP-40, 0.15 mM spermine (Sigma-Aldrich, St. Louis, MO, USA), 0.5 mM spermidine(Sigma-Aldrich), followed by incubation on ice for 5 min. Nuclei were pelleted by centrifuge at 5000 rpm for 3 min at 4 ˚C and resuspended with MNase digestion buffer without CaCl_2_ (10 mM Tris-HCl (pH 7.4), 15 mM NaCl, 60 mM KCl, 0.15 mM spermine, 0.5 mM spermidine). After centrifugation, the nuclei were resuspended with MNase digestion buffer supplemented with CaCl_2._ Then, half of the each sample was treated MNase with 5 unit/sample and the other half of that was treated digestion buffer, and samples were incubated at 37 ˚C for 1 min. The digestion reaction was stopped by addition of stop solution (100 mM EDTA/10 mM EGTA (pH 8.1) in 10 mM Tris-HCl (pH 7.4)). RNaseA (10 μg/sample) and proteinase K (100 μg/sample) were added and samples were incubated at 37 ˚C overnight. DNA, purified by Phenol/chloroform/isoamyl alcohol extraction, was analyzed by q-PCR using primer pairs covering -360/-155 of the PPARγ2 promoter, and -273/-203 of the GAPDH promoter. Chromatin accessibility was calculated as described in Fig. 4 legend.

### Chromatin immunoprecipitation (ChIP)

ChIP was performed using a commercial kit (Millipore, Bedford, MA, USA). In brief, cells were crosslinked with 1% formaldehyde followed by addition of 125 mM glycine. After washing, cells were sonicated and immunoprecipitated with normal serum, anti-TonEBP, anti-H3K9me2 (Abcam, Cambridge, MA, USA), and anti-C/EBPβ (Abcam) antibodies at 4 ˚C overnight. After elution and reverse crosslinking the antibody/DNA complexes, DNA was purified by DNA purification kit (Qiagen, Hilden, Germany) and analyzed by q-PCR using primer pairs covering specific region of the PPARγ2 promoter in duplicates. Primer pairs for q-PCR covered -360/-155 of the PPARγ2 promoter. Data was shown as amount of DNA relative to input.

### Statistical analysis

Data are presented as means + SD. Differences between groups were analyzed by student’s *t*-test, and statistically significance was considered at *p < 0.05.

## Additional Information

**How to cite this article**: Lee, J.H. *et al.* TonEBP suppresses adipogenesis and insulin sensitivity by blocking epigenetic transition of PPARγ2. *Sci. Rep.* **5**, 10937; doi: 10.1038/srep10937 (2015).

## Supplementary Material

Supplementary Information
