# Supplementary material for: TonEBP suppresses adipogenesis and insulin sensitivity by blocking epigenetic transition of PPARγ2
Source: Sci Rep. 2015 Jun 4;5:10937. doi: 10.1038/srep10937 (PMC4455245; doi:10.1038/srep10937)
Supplement: Supplementary Information [file srep10937-s1.pdf]

TonEBP suppresses adipogenesis and insulin sensitivity by blocking epigenetic transition of PPARγ2

Jun Ho Lee, Hwan Hee Lee, Byeong Jin Ye, Whaseon Lee-Kwon, Soo Youn Choi*, and Hyug Moo Kwon*.

School of Life Sciences, Ulsan National Institute of Science and Technology, Ulsan, Republic of Korea

* Address correspondence to H.M. Kwon (hmkwon@unist.ac.kr) or S.Y. Choi (sychoi@unist.ac.kr).

Building 104, Room 709

School of Life Sciences

Eoyang-eup, Ulju-gun

Ulsan 689-798

Republic of Korea

Tel: +82-217-52-2633

Supplementary Figures

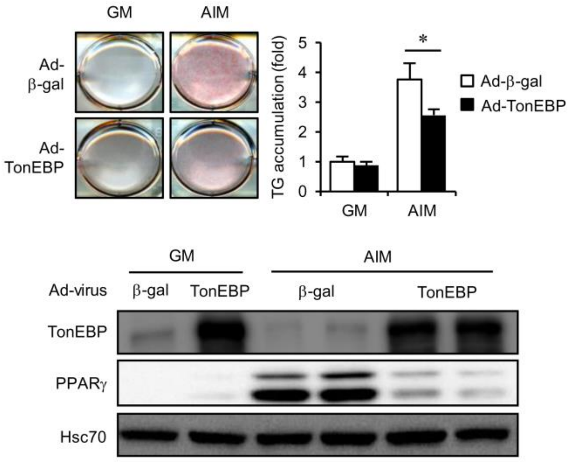

Supplementary Figure S1 (related to Figure 1). TonEBP overexpression suppresses adipogenesis and PPARγ expression.

3T3-L1 cells were infected with adenovirus expressing TonEBP or β-galactosidase (β-gal) as indicated, followed by a 6 day culture in AIM. The cells were immunoblotted for TonEBP and Hsc70, and stained with Oil-Red-O and the stain was extracted and measured to assess intracellular TG. Relative intracellular TG was represented as Mean + SD, n = 4. *$p < 0.05$.

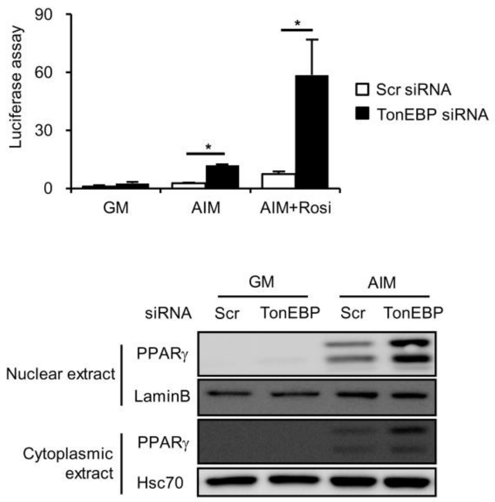

Supplementary Figure S2 (Related Figure 2). TonEBP suppresses PPARγ transcription activity.

3T3-L1 transfected with scrambled (Scr, open bars) or TonEBP-targeted siRNA (filled bars) were cultured for 4 days in GM or AIM without or with rosiglitazone. The cells were transfected with PPAR-responsive element (PPRE) reporter plasmid and luciferase activity was measured. The cells were fractionated into nuclear and cytoplasmic fractions and immunoblotted for PPARγ, lamin B and Hsc70.

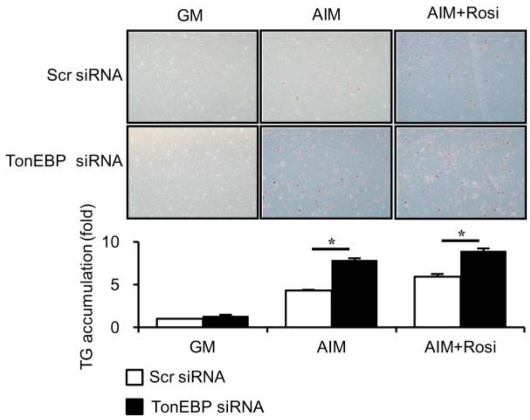

Supplementary Figure S3 (Related to Figure 1 and Figure 2). TonEBP suppresses adipogenesis from human bone marrow-derived mesenchymal stem cells.

Cells were transfected with scrambled (open bars) or TonEBP targeted siRNA (filled bars) and cultured in AIM as described in Fig 1. Mean + SD, n = 4. $^*p < 0.05$

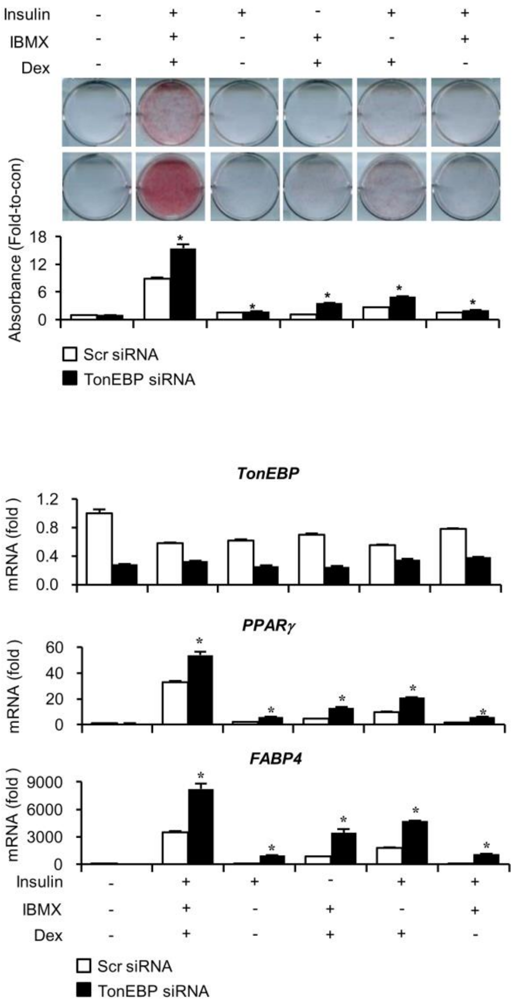

Supplementary Figure S4 (Related to Figure 4). TonEBP suppresses adipocytes differentiation independent of insulin, IBMX, and dexamethasone (Dex).

3T3-L1 transfected with scrambled or TonEBP-targeted siRNA were cultured for 6 days in AIM or various AIM without insulin, IBMX, or Dex as indicated. Images of Oil-Red-O stained cells and spectrophotometric quantification are shown. Quantitative RT-PCR analyses of mRNA for TonEBP, PPARγ, and FABP4 are shown. Mean + SD, n = 4. *$p < 0.05$ vs. corresponding scr siRNA.

Supplementary Table

Supplementary Table S1.

TonEBP	5’-AAGCAGCCACCACCAAACATGA-3’ and 5’-AAATTGCATGGGCTGCTGCT-3’
C/EBPβ	5’-AGCTGAGCGACGAGTACAAGAT-3’ and 5’-TTGAACAAGTTCCGCAGGGT-3’
C/EBPα	5’-GTCACTGGTCAACTCCAGCAC-3’ and 5’-CAAGAACAGCAACGAGTACCG-3’
PPARγ1	5’-AGGAGAAGTCACACTCTGACAGGA-3’ and 5’-TCAGTGGTTCACCGCTTCTT-3’
PPARγ2	5’-TTCGCTGATGCACTGCCTATGA-3’ and 5’-AAGGAATGCGAGTGGTCTTCCA-3’
PPARγ	5’-TCTTAACTGCCGGATCCACAA-3’ and 5’-GCCCAAACCTGATGGCATT-3’
FABP4	5’-TGGAAGCTTGTCTCCAGTGA-3’ and 5’-AATCCCCATTTACGCTGATG-3’
SREBP-1c	5’-GCCGTGGTGAGAAGCGCACAGCCC-3’ and 5’-CAAGACAGCAGATTTATTCAGCTTTGC-3’
SCD1	5’-TTCTTGCGATACACTCTGGTGC-3’ and 5’-CGGGATTGAATGTTCTTGTCGT-3’
ACC	5’-CTGACGTATACTGAACTGGTGTTGGATG-3’ and 5’-TTTCCAGGCTACCATGCCAATCTC-3’
Adiponectin	5’-TGGAATGACAGGAGCTGAAGG3’ and 5’-ACACTGAACGCTGAGCGATACACA-3’
Glut4	5’-CAATGGTTGGGAAGGAAAAGGGCTA-3’ and 5’-GTAGGCGCCAATGAGGAACCGTC-3’
TNFα	5’-TGGGACAGTGACCTGGACTGT-3’ and 5’-TTCGGAAAGCCCATTTGAGT-3’
Leptin	5’-GGGCTTCACCCCATTCTGA-3’ and 5’-TGGCTATCTGCAGCACATTTTG-3’
Resistin	5’-TCGTGGGACATTCGTGAAGA-3’ and 5’-GGGCTGCTGTCCAGTCTATCC-3’
MCP-1	5’-AACTGCATCTGCCCTAAGGT-3’ and 5’-AGTGCTTGAGGTGGTTGTGGAA-3’
